# Association between *FTO* polymorphism and COVID-19 mortality among older adults: A population-based cohort study

**DOI:** 10.1016/j.ijid.2024.107232

**Published:** 2024-11

**Authors:** Jaroslav A. Hubacek, Nadezda Capkova, Martin Bobak, Hynek Pikhart

**Affiliations:** 1 Institute of Clinical and Experimental Medicine, Experimental Medicine Centre, Prague, Czech Republic; 2 Charles University, Third Department of Internal Medicine, First Faculty of Medicine, Prague, Czech Republic; 3 National Institute of Public Health, Prague, Czech Republic; 4 University College London, Institute of Epidemiology and Health Care, London, United Kingdom; 5 Masaryk University, RECETOX, Faculty of Science, Brno, Czech Republic

**Keywords:** COVID-19, SARS-CoV-2, FTO, Mortality, Polymorphism

## Abstract

**Objectives:**

COVID-19 caused a global pandemic with millions of deaths. Fat mass and obesity-associated gene (*FTO*) (alias m^6^A RNA demethylase) and its functional rs17817449 polymorphism are candidates to influence COVID-19-associated mortality since methylation status of viral nucleic acids is an important factor influencing viral viability.

**Methods:**

We tested a population-based cohort of 5233 subjects (aged 63-87 years in 2020) where 70 persons died from COVID-19 and 394 from other causes during the pandemic period.

**Results:**

The frequency of GG homozygotes was higher among those who died from COVID-19 (34%) than among survivors (19%) or deaths from other causes (20%), *P* <0.005. After multiple adjustments, GG homozygotes had a higher risk of death from COVID-19 with odds ratio = 2.01 (95% confidence interval; 1.19-3.41, *P* <0.01) compared with carriers of at least one T allele. The *FTO* polymorphism was not associated with mortality from other causes.

**Conclusions:**

Our results suggest that *FTO* variability is a significant predictor of COVID-19-associated mortality in Caucasians.

## Highlights

• Fat mass and obesity-associated gene (*FTO*) is a significant epigenetic modifier.
• We included 5180 subjects (70 died from COVID-19 and 341 from other causes).
• GG homozygotes had a higher risk of death from COVID-19 (odds ratio = 2.01; *P* <0.01).
• Mortality from other causes was independent of FTO polymorphism (odds ratio = 0.99).
• FTO rs17817449 single nucleotide polymorphism is associated with the risk of death among COVID-19 patients.

## Introduction

SARS-CoV-2 infection caused the global pandemic of COVID-19 disease with nearly 775 million cases and 7 million cumulative deaths worldwide. The Czech Republic was among the countries most affected [1], with more than 4.5 million confirmed cumulative cases and almost 43,500 deaths (//covid19.who.int, accessed March 07, 2024).

Several factors, such as hypertension, male sex, non-white ethnicities, or diabetes and obesity, have been found to significantly increase the risk of infection and less favorable prognosis [2,3]. Importantly, studies with different protocols (both case-control studies and genome-wide association studies) have identified dozens of genes/loci that correlate with susceptibility to the SARS-CoV-2 infection and/or COVID-19 severity (for review see [[4], [5], [6]]). Host genomic biomarkers may explain inter-individual variability in the risk of infection and progression to severe COVID-19 clinical manifestations/mortality. Genetic variants may affect viral entry survival and replication, and modulate antiviral immunity, but the mechanistic link to COVID-19 remains unclear.

Among the first genetic variants associated with COVID-19 were polymorphisms within the angiotensin-converting enzyme (ACE1*)*, apolipoprotein E (APOE*),* and chemokine receptor 5 (*CCR5)* genes. Blood group A and various human leucocyte antigen (HLA) types are widely recognized as being risky. Variants transferred to the modern human genome from Neanderthals (at oligoadenylate synthetase [*OAS1*] and leucine zipper transcription factor-like 1 [*LZTFL1*]), are among the strongest predictors of disease severity. Finally, the last evidence also pointed to the importance of host variants within the interferon-induced antiviral factor (*IFITM3),* angiotensin 2 receptor (*AGTR2*), and trans-membrane protease serine 2 (*TMPRSS2)*. It is important to note that the associations of common polymorphism with clinical outcomes are relatively weak, with odds ratios mostly between 1.1 and 1.7, although some rare variants (several interferon genes) may have stronger effects [7].

Fat mass and obesity-associated gene *(FTO)* (m6A [N6-methyladenosine] demethylase; Online Mendelian Inheritance in Man acc. No. 610966) would seem a promising candidate to influence infection susceptibility and severity since variability within this gene (a cluster of tagging single nucleotide polymorphisms [SNPs] within the first intron of the gene with a very high degree of linkage disequilibrium) has been shown to be associated with obesity [8,9] and type II diabetes mellitus [10]. Subsequent studies broadened/widened the importance of this gene in the determination of renal failure [11], myocardial infarction [12], several types of cancer [13] or even osteoporosis [14].

This wide spectrum of heterogeneous clinical complications associated with *FTO* variability could have an epigenetic background. Indeed, *FTO* has been rapidly recognized as an epigenetic modifier both at the DNA [15] and at RNA [16] level. *FTO* m6A demethylase activity has been described as an important factor influencing viral viability and replication [[17], [18], [19]].

In the past, variants within the *FTO* have also been associated with infection susceptibility (albeit not virus-cased), namely tuberculosis [20]. No detailed genetic-epidemiology analyses have been performed so far in the field of virus infection, despite the fact, that the effect of *FTO* on viral viability has recently been suggested [[17], [18], [19]].

In this report, we investigated the potential role of variability in the *FTO* 1^st^ intron tagging SNPs (rs17817449, NG_012969.1:g.80493T>G) in COVID-19 mortality in a population-based cohort of subjects followed up during the COVID-19 pandemic for cause-specific mortality.

## Methods

### Data collection

Subjects are from the Czech branch of the HAPIEE (Health Alcohol and Psychological Factors in Eastern Europe) study. Overall, 8857 men and women aged 45-69 years in 2002 were examined in the baseline survey in 2002-2005 [21]. At baseline, participants completed extensive questionnaires on their health, lifestyle and health behaviors, and socio-economic and psychosocial circumstances and were invited to physical examination on a separate occasion, during which they a provided a blood sample. A total of 6552 persons provided blood samples which allowed successful extraction of DNA.

Deaths in the cohort were identified by linkage with the national mortality register from 2002 until the end of June 2022. Deaths in the period 1 March 2020-30 June 2022 were used in this report; these 2 years cover the most severe pandemic period, while the mortality data from the second half of 2022 are not yet available.

This report is based on 5233 subjects with DNA samples who were alive on 1 March 2020. During ∼2 years of interest (1 March 2020-30 June 2022) 70 subjects died from COVID-19 (ICD-10 code U07.1) and a further 394 subjects died from other causes.

### DNA analyses

DNA has been isolated from whole ethylenediaminetetraacetic acid tetrasodium (EDTA) blood, as described in detail by Miller et al*.* [22]. Tagging *FTO* first intron polymorphism rs17817449 has been analyzed by polymerase chain reaction-RFLP (restriction fragment lenght polymorphism) and kompetitive allele specific PCR (KASP^TM^) methods as described in detail elsewhere [23,24].

### Statistical analysis

Cross-tabulations and multinomial logistic regression were used to assess differences in *FTO* genotype between survivors vs those who died during the pandemic period of our interest. Baseline anthropometric measurements, socio-demographic indicators, and health variables were used as covariates, including diabetes, age, sex, history of ever smoking prevalence, body mass index, education, and marital status. STATA statistical software was used.

## Results

There were 464 deaths among eligible participants during the two main pandemic years between 1 March 2020 and 30 June 2022; 70 of these deaths were coded as COVID-19. Descriptive baseline characteristics of the study subjects by survival status are summarized in Table 1. There were some important differences between survivors and those who died from COVID-19 and from other causes. Survivors were younger, more likely to be female, had lower body mass index, and were less likely to be married, to report diabetes, and to have higher education.

**Table 1:** Descriptive characteristics of subjects in the study.

	Survivors	COVID-19 deaths	Other deaths	*P*^a^
N	4769	70	394	
Age on 01/2020, mean	73.0	78.7	78.4	<0.001
Sex, %				
Males	40.5	64.3	57.9	<0.001
Females	59.5	35.7	42.1	
Body mass index (kg/m^2^), mean	27.8	29.9	28.8	<0.001
Smoking, %	21.0	13.2	24.4	0.08
Diabetes, %	7.8	14.3	15.0	<0.001
Education, %				
Below secondary	46.3	63.8	53.4	0.003
Secondary	39.1	26.1	32.6	
University	14.6	10.1	14.0	
Married, %	75.2	85.7	82.0	0.002

a *P*-value for difference between groups (chi-square or analysis of variance as appropriate).

The distribution of individual *FTO* genotypes in the entire cohort (Table 2) was similar to the other Caucasian populations (42.7% of the G allele within the HAPIEE study and 40.4% of the G allele within pooled Caucasian studies, according the http://www.ncbi.nlm.gov/snp/rs17817449) with apparent excess of GG homozygotes among participants who have died from COVID-19. These differences were similar among both sexes.

**Table 2:** FTO polymorphism distribution by survival status and sex.

(a) Total population						
*FTO*	Survivors		COVID-19 deaths		Other deaths	
rs17817449	N	%	N	%	N	%
	4769		70		394	
TT	1593	33.4	19	27.1	124	31.5
TG	2274	47.7	27	38.6	191	48.5
GG	902	18.9	24	34.3	79	20.0

(b) Males						
*FTO*	Survivors		COVID-19 deaths		Other deaths	
rs17817449	N	%	N	%	N	%

	1929		45		228	
TT	633	32.8	13	28.9	70	29.7
TG	923	47.8	17	37.8	108	47.9
GG	373	19.3	15	33.3	50	22.4

(c) Females						
*FTO*	Survivors		COVID-19 deaths		Other deaths	
rs17817449	N	%	N	%	N	%

	2840		25		166	
TT	960	33.8	6	24.0	54	32.9
TG	1,351	47.6	10	40.0	83	49.7
GG	529	18.6	9	36.0	29	17.4

The *FTO* SNP was not significantly associated with all-cause mortality during the pandemic, regardless of the type of comparison (*P* = 0.15 for GG vs GT vs TT comparison). When mortality was analyzed by the cause of death, the G allele of the rs17817449 polymorphism was associated with COVID-19 mortality in a recessive manner (there was no association with GT heterozygotes). GG homozygotes were significantly more frequent among those who died from COVID-19 compared with survivors (*P* = 0.005).

The *FTO* GG genotype remained associated with an increased risk of COVID-19 mortality both before and after multiple adjustments, with an adjusted odds ratio of 2.01 (95% confidence interval; 1.19-3.41, *P* <0.01) compared with survivors (Table 3). No significant association was observed between mortality from other causes and *FTO* genotypes regardless of the type of comparison and adjustment applied.

**Table 3:** Odds ratios (95% confidence interval) for mortality by FTO genotype (GG homozygotes vs others).

*FTO* rs17817449	Unadjusted	Model 1	Model 2	Model 3
	**COVID-19 deaths**			
T allele carriers	1.00	1.00	1.00	1.00
GG homozygotes	2.24 (1.36-3.68)	2.20 (1.33-3.64)	2.08 (1.25-3.45)	2.01 (1.19-3.41)
*P*	0.002	0.002	0.005	0.01

	**Non-COVID-19 deaths**			

T allele carriers	1.00	1.00	1.00	1.00
GG homozygotes	1.08 (0.83-1.39)	1.06 (0.81-1.38)	1.03 (0.79-1.35)	0.99 (0.75-1.30)
*P*	0.58	0.69	0.80	0.93

Model 1: adjusted for age, sex, and diabetes.

Model 2: adjusted for age, sex, body mass index, and diabetes.

Model 3: adjusted for age, sex, body mass index, diabetes, smoking, education, and marital status.

## Discussion

To our best knowledge, this study is the first to associate the risk of COVID-19 death with the variability within the RNA demethylase—*FTO*.

The *FTO* genotype has been previously associated with the risk of several non-communicable diseases (such as obesity, renal failure, myocardial infarction, or diabetes).

The association between *FTO* and the risk of death from COVID-19 is biologically plausible, given the recently identified functions of this gene. The *FTO* gene encodes a protein that is an important epigenetic modulator—it encodes a nucleic acid demethylase, that catalysis the demethylation both of the DNA [15] and RNA [16].

FTO is an m^6^A (N6-methyladenosine) demethylase—it catalyzes the most common post-transcriptional modification of all RNA molecules, including viral RNA [25]. m^6^A modification is dependent on methyltransferases and proteins recognized as m^6^A writers, readers, and erasers [26]. FTO (together with ALKBH5 and ALKBH3) is one of the most potent m^6^A erasers—it catalyzes the demethylation of modified RNA [25]. This modification significantly influences the replication and assembly of viral particles.

RNA methylation is one of the most important post-transcriptional regulation, not only in vertebrates [27], but it is also a key effector of viral RNA replication [26]. m^6^A is the most common type of viral RNA modification, regulating viral genomic RNA synthesis, its stability, and potentially also translation. In fact, m^6^A present in SARS virus RNA affects virus replication [28]. It has been described that SARS-CoV-2 RNA is modified by m^6^A machinery [29]. SARS-CoV-2 infection decreased the FTO expression within the cell line, and in a separate experiment, the knockdown of FTO resulted in a significant increase in viral titer. This clearly confirms the importance of FTO in viral replication. It can be speculated that in the presence of the functional FTO protein, increased demethylation of m^6^A leads to the disruption of the SARS-CoV-2 viability, lower numbers of virus particles, and consequently probably leads to the milder disease course.

Importantly, *FTO* expression levels are associated with demethylation efficacy. Using the human cell models, it has been shown that *FTO* overexpression leads to a reduction of m^6^A content, and *FTO* knockdown leads to an increase of m^6^A content in RNA [16]. Unfortunately, no consistent data are available on *FTO* expression according to first intron gene variability. Several reports have examined tissue *FTO* expression in dependence on the first intron SNPs. However, the results are inconsistent (probably mainly as a consequence of the fact that samples from different tissues have been analyzed and the body mass index values of the examined subjects were often extreme), with some suggesting lower expression in risky homozygotes [30].

SARS-CoV-2 virus RNA contains a high proportion of m6A modifications [28] and retains at least one predicted strong FTO binding site [19]—FTO is one of the RNA binding proteins that are critical host factors for SARS-CoV-2 infection. Functional *in silico* mutation stimulation identified FTO as a potential biomarker of COVID-19.

It has been proved, that selective inhibition of the FTO activity by Rhein causes a significant reduction of SARS-CoV-2 infectivity and, at high concentrations, could completely block the infection [17]. Importantly, it has been emphasized, that the concentrations used showed no signs of cytotoxicity and could therefore be used in a potential treatment of COVID-19.

Interestingly, genome-wide association studies [31,32], or whole-genome sequencing [33] failed to find an *FTO* as a candidate gene for COVID-19 severity. This discrepancy may be explained by the fact that while the abovementioned studies generally included critically ill and hospitalized subjects, they did not distinguish between COVID-19 survivors and non-survivors. Unfortunately, we do not have data on COVID-19 incidence in the cohort to assess this potential explanation.

Our study has several strengths. It is one of the few relatively larger cohort studies reporting genetic determination of COVID-19 mortality, susceptibility, and severity. The cohort includes an ethically and genetically homogeneous population from a geographically relatively small region. Thus, the ethnicity or differences in population-based measures against the spread of the epidemic would play only a minor role in the outcome. Importantly we have got also information about the mortality from other causes, which is extremely important given that *FTO* has been suggested to have also effect on general mortality [34], an observation that we have not confirmed. Finally, about a third of the study sample has confirmed *FTO* genotyping results by two independent methods [24], minimizing the possibility of false positive results.

However, there are several limitations. First, the number of COVID-19 deaths was relatively small, although the proportion of excess deaths in the cohort was consistent with national data. While the possibility of a false positive finding is low, it cannot be excluded. Second, the coding of COVID-19 as the main cause of death is not absolutely reliable and some deaths may have been miscoded. But, if this was the case, and if the effect of *FTO* is specific to COVID-19, our results would be underestimates. Finally, as we do not have a confirmatory population, our result requires an independent confirmation.

We conclude that genetic variability within the *FTO*, m6A demethylase, may be an important predictor of COVID-19-associated mortality. Further roles of *FTO* variability in COVID-19 etiology, such as the risk of infection *per se* or association with less severe illness need to be further investigated.

## Declarations of competing interest

The authors have no competing interests to declare.

## References

[1] Tuček M, Vaněček V. (2022). COVID-19 in the Czech Republic 2020 and 2021: comparative analysis of probable work-related transmission of the coronavirus SARS-CoV-2. Cent Eur J Public Health.

[2] Chams N, Chams S, Badran R, Shams A, Araji A, Raad M (2020). COVID-19: a multidisciplinary review. Front Public Health.

[3] Dessie ZG, Zewotir T. (2021). Mortality-related risk factors of COVID-19: a systematic review and meta-analysis of 42 studies and 423,117 patients. BMC Infect Dis.

[4] Hubacek JA. (2021). Effects of selected inherited factors on susceptibility to SARS-CoV-2 infection and COVID-19 progression. Physiol Res.

[5] Gupta K, Kaur G, Pathak T, Banerjee I. (2022). Systematic review and meta-analysis of human genetic variants contributing to COVID-19 susceptibility and severity. Gene.

[6] Delanghe JR, Speeckaert MM (2022). host. Host polymorphisms and COVID-19 infection. Adv Clin Chem.

[7] Zhang Q, Bastard P, Human Genetic Effort COVID, Cobat A, Casanova JL (2022). Human genetic and immunological determinants of critical COVID-19 pneumonia. Nature.

[8] Dina C, Meyre D, Gallina S, Durand E, Körner A, Jacobson P (2007). Variation in FTO contributes to childhood obesity and severe adult obesity. Nat Genet.

[9] Frayling TM, Timpson NJ, Weedon MN, Zeggini E, Freathy RM, Lindgren CM (2007). A common variant in the FTO gene is associated with body mass index and predisposes to childhood and adult obesity. Science.

[10] Scott LJ, Mohlke KL, Bonnycastle LL, Willer CJ, Li Y, Duren WL (2007). A genome-wide association study of type 2 diabetes in Finns detects multiple susceptibility variants. Science.

[11] Hubacek JA, Viklicky O, Dlouha D, Bloudickova S, Kubinova R, Peasey A (2012). The FTO gene polymorphism is associated with end-stage renal disease: two large independent case-control studies in a general population. Nephrol Dial Transplant.

[12] Lappalainen T, Kolehmainen M, Schwab US, Tolppanen AM, Stančáková A, Lindström J (2011). Association of the FTO gene variant (rs9939609) with cardiovascular disease in men with abnormal glucose metabolism–the Finnish Diabetes Prevention Study. Nutr Metab Cardiovasc Dis.

[13] Lan N, Lu Y, Zhang Y, Pu S, Xi H, Nie X (2020). FTO - A common genetic basis for obesity and cancer. Front Genet.

[14] Usategui-Martín R, Pérez-Castrillón JL, Briongos-Figuero L, Abadía-Otero J, Lara-Hernandez F, García-Sorribes S (2022). Genetic variants in obesity-related genes and the risk of osteoporotic fracture. The Hortega follow-up study. Front Biosci (Landmark Ed).

[15] Gerken T, Girard CA, Tung Y-CL, Webby CJ, Saudek V, Hewitson KS (2007). The obesity-associated FTO gene encodes a 2-oxoglutarate-dependent nucleic acid demethylase. Science.

[16] Jia G, Fu Y, Zhao X, Dai Q, Zheng G, Yang Y (2011). N6-methyladenosine in nuclear RNA is a major substrate of the obesity-associated FTO. Nat Chem Biol.

[17] Zannella C, Rinaldi L, Boccia G, Chianese A, Sasso FC, De Caro F (2021). Regulation of m6A methylation as a new therapeutic option against COVID-19. Pharmaceuticals (Basel).

[18] Moon JS, Lee W, Cho YH, Kim Y, Kim GW. (2024). The significance of N6-methyladenosine RNA methylation in regulating the hepatitis B virus life cycle. J Microbiol Biotechnol.

[19] Horlacher M, Oleshko S, Hu Y, Ghanbari M, Cantini G, Schinke P (2023). A computational map of the human-SARS-CoV-2 protein-RNA interactome predicted at single-nucleotide resolution. NAR Genom Bioinform.

[20] Feng Y, Wang F, Pan H, Qiu S, Lü J, Wu L (2014). Obesity-associated gene FTO rs9939609 polymorphism in relation to the risk of tuberculosis. BMC Infect Dis.

[21] Peasey A, Bobak M, Kubinova R, Malyutina S, Pajak A, Tamosiunas A (2006). Determinants of cardiovascular disease and other non-communicable diseases in Central and Eastern Europe: rationale and design of the HAPIEE study. BMC Public Health.

[22] Miller SA, Dykes DD, Polesky HF. (1988). A simple salting out procedure for extracting DNA from human nucleated cells. Nucleic Acids Res.

[23] Hubacek JA, Bohuslavova R, Kuthanova L, Kubinova R, Peasey A, Pikhart H (2008). The FTO gene and obesity in a large Eastern European population sample: the HAPIEE study. Obesity (Silver Spring).

[24] Hubáček JA, Pikhart H, Peasey A, Kubínová R, Bobák M. (2015). Nobody is perfect: comparison of the accuracy of PCR-RFLP and KASP™ method for genotyping. ADH1B and FTO polymorphisms as examples. Folia Biol (Praha).

[25] Molinie B, Wang J, Lim KS, Hillebrand R, Lu ZX, Van Wittenberghe N (2016). m(6)A-LAIC-seq reveals the census and complexity of the m(6)A epitranscriptome. Nat Methods.

[26] Chen Y, Wang W, Zhang W, He M, Li Y, Qu G (2023). Emerging roles of biological m6A proteins in regulating virus infection: a review. Int J Biol Macromol.

[27] Zhou Y, Kong Y, Fan W, Tao T, Xiao Q, Li N (2020). Principles of RNA methylation and their implications for biology and medicine. Biomed Pharmacother.

[28] Liu J, Xu YP, Li K, Ye Q, Zhou HY, Sun H (2021). The m6A methylome of SARS-CoV-2 in host cells. Cell Res.

[29] Zhang X, Hao H, Ma L, Zhang Y, Hu X, Chen Z (2021). Methyltransferase-like 3 modulates severe acute respiratory syndrome coronavirus-2 RNA N6-methyladenosine modification and replication. mBio.

[30] Doaei S, Kalantari N, Keshavarz Mohammadi N, Izadi P, Gholamalizadeh M, Eini-Zinab H (2019). The role of FTO genotype in the association between FTO gene expression and anthropometric measures in obese and overweight adolescent boys. Am J Mens Health.

[31] COVID (2021). Mapping the human genetic architecture of COVID-19. Nature.

[32] Pairo-Castineira E, Rawlik K, Bretherick AD, Qi T, Wu Y, Nassiri I, et al. GWAS and meta-analysis identifies 49 genetic variants underlying critical COVID-19. Nature 2023;617:764–8. 10.1038/s41586-023-06034-3. Erratum in: Nature 2023;619:E61. 10.1038/s41586-023-06034-3. PMC10208981 37198478

[33] Kousathanas A, Pairo-Castineira E, Rawlik K, Stuckey A, Odhams CA, Walker S (2022). Whole-genome sequencing reveals host factors underlying critical COVID-19. Nature.

[34] Zimmermann E, Kring SII, Berentzen TL, Holst C, Pers TH, Hansen T (2009). Fatness-associated FTO gene variant increases mortality independent of fatness - in cohorts of Danish men. PLoS One.

